# Slow reduction of IP-10 Levels predicts HBeAg seroconversion in chronic hepatitis B patients with 5 years of entecavir treatment

**DOI:** 10.1038/srep37015

**Published:** 2016-11-15

**Authors:** Renyong Guo, Hejun Mao, Xiao Hu, Nengneng Zheng, Dong Yan, Jianqin He, Jiezuan Yang

**Affiliations:** 1 Department of Laboratory Medicine, First Affiliated Hospital, College of Medicine, Zhejiang University; Key Laboratory of Clinical In Vitro Diagnostic Techniques of Zhejiang Province, Hangzhou, China; 2 Maternity and Child Health Care Hospital of Xiacheng District, Hangzhou, China; 3 Department of Gynecology and Obstetrics, Tongde Hospital of Zhejiang Province, Hangzhou, China; 4 State Key Laboratory for Diagnosis and Treatment of Infectious Diseases; Collaborative Innovation Center for Diagnosis and Treatment of Infectious Diseases; First Affiliated Hospital, College of Medicine, Zhejiang University, Hangzhou, China

## Abstract

The aim of this study was to determine the correlation between dynamic changes in serum cytokine/chemokine expression levels in response to entecavir (ETV) treatment and HBV e antigen (HBeAg) seroconversion in patients with chronic hepatitis B (CHB). Four cytokines (interleukin [IL]-4, IL-6, IL-8, and interferon-γ) and five chemokines (macro-phage inflammatory protein [MIP]-1α, MIP-1β, platelet derived growth factor-BB, and interferon-inducible protein 10 [IP-10]) before ETV therapy and at 3, 6, 12, 24, 36 and 60 months during therapy in 105 CHB patients were analyzed. The results showed that the low decrease rate of IP-10 levels after 1 year of ETV treatment was an independent predictor of HBeAg seroconversion at year 5 (Hazard ratio = 0.972). The area under the receiver operating characteristic curves for the decrease rate of IP-10 levels after 1 year of treatment to discriminate a year-5 HBeAg seroconversion was 0.752 (p = 0.005). The results indicate that higher IP-10 level at year one of ETV treatment is associated with an increased probability of HBeAg seroconversion. Quantification of IP-10 during ETV treatment may help to predict long-term HBeAg seroconversion in patients with CHB.

Despite the availability of highly effective and safe vaccination for more than 20 years, hepatitis B virus (HBV) chronic infection continues to play an important role in human disease, infecting nearly 350 million people worldwide. Complications are associated with this disease including liver cirrhosis, hepatocellular carcinoma, and hepatic failure, and contributing to an estimated 800,000 premature deaths annually^1^. Recently, the management of chronic hepatitis B (CHB) has been revolutionized by the introduction of long-term nucleoside analogue therapy, which has been proven to reduce liver related complications and improve clinical outcomes^2^. Entecavir (ETV), a nucleoside analogue used for the treatment of CHB, has been shown to achieve favourable outcomes based on direct measures of antiviral efficacy and on several clinical experimental results, including HBV-DNA detection, alanine aminotransferase (ALT) and HBV serologic tests, in both HBV e antigen (HBeAg)-positive and HBeAg-negative CHB patients^3^,^4^. Furthermore, ETV has a high genetic barrier to resistance with only minimal problems associated with resistance in treatment-naïve and treatment-experienced patients^5^. In addition, treatment with ETV can improve the outcome of liver fibrosis and cirrhosis in patience with CHB^6^. Nevertheless, to our knowledge, the therapeutic effects and immunoregulatory functions of ETV have not been full studied in CHB patients.

HBeAg status is a critical parameter for monitoring and/or following-up of both CHB progression and treatment. Positive HBeAg status is indicative of viral replication and increases in the risk of developing liver cirrhosis and hepatocellular carcinoma^7^. Indeed, HBeAg seroconversion is considered one of the most important surrogate markers for assessing the durability and efficacy of antiviral therapy in CHB patients with HBeAg-positive^8^. However, the exact mechanisms and factors that contribute to HBeAg seroclearance and HBeAb production are not well described. Cytokines not only inhibit viral replication, but determine the predominant pattern of the host immune response^9^,^10^. Chemokines, a family of small cytokines, direct the migration of circulating leukocytes to sites of inflammation or injury, and enhance the phagocytic power of the inflammatory cells^11^,^12^. As the control of cytokine secretion is highly complex and the interactions between cytokines and their receptors are widespread throughout multiple regulatory networks, screening for multiple biomarkers may elucidate the immunopathogenesis of HBV infection and predict responses to antiviral therapy. Indeed, a previous study reported that the T-cell response and circulating cytokine profile were associated with viral replication and liver function in CHB^13^. Interleukin (IL)-10, IL-12, IL-21 have important roles during the immune clearance phase and hence are associated with HBeAg seroconversion^14^,^15^. However, the relationship between multiple cytokines and chemokines and responses to ETV therapy in patients with CHB has not been fully elucidated in the Chinese population.

In this study, we aimed to investigate the dynamic changes in the host immune response by examining serum cytokine and chemokine levels in response to ETV treatment and to determine which cytokines and chemokines are related to HBeAg seroconversion in patients with CHB.

## Results

### Baseline clinical characteristics of patients

A total of 105 CHB patients (88.2%) completed 5 years of ETV treatment and 14 patients (11.8%) withdrew prematurely; 2.5% withdrew consent, 5.0% deviated from protocol, and 4.2% was lost to follow-up. Among the 105 patients, 71 (67.6%) underwent a liver biopsy, 13 (18.3%) had cirrhosis before treatment, 48 (45.7%) attained HBeAg seroconversion at year 5 post-ETV treatment. Demographic and clinical data of CHB patients and healthy controls are shown in Table 1. All patients reached normal ALT, AST, TB, and undetectable HBVDNA after 5 years of ETV treatment. The patients and healthy controls had similar age and gender distributions, while higher baseline levels of TB, ALT, AST, along with a lower level of ALB, were found in CHB patients when compared to those in healthy controls. Levels of HBsAg, HBeAg, and HBV DNA at baseline were significantly higher in patients without HBeAg seroconversion than in those with HBeAg seroconversion. No significant differences were found between patient groups with regard to age, sex, HBV genotype, Metavir score, the number of liver biopsy or cirrhosis, TB, ALB, AST, or ALT.

### Serum cytokine and chemokine profiles in patients with CHB

Levels of various serum cytokines/chemokines in patients and in healthy controls were measured. Baseline serum concentrations of all nine cytokines/chemokines were significantly higher in patients with CHB than in healthy controls (p < 0.05). When we subdivided patients into HBeAg seroconversion and non-HBeAg seroconversion, however, no significant differences in the baseline levels of any cytokine/chemokine were found between the two patient groups (Supplementary Table 1).

The association of clinical parameters and cytokines/chemokines with hepatic fibrosis before ETV therapy was further evaluated. Compared with the patients without cirrhosis (Metavir score ≤ 3, n = 58), patients with cirrhosis (Metavir score = 4, n = 13) had markedly high levels of AST and PDGF-BB. Marginal differences of IL-6 and HBsAg were also observed between the two patient groups (Supplementary Table 2).

### Correlation between cytokines/chemokines and biochemical parameters

Several clinical parameters at baseline were examined for their correlation with serum cytokines/chemokines in patients with CHB. Nine serum cytokines/chemokines were significantly positively correlated with values for ALT (p < 0.05) and AST (p < 0.05). MIP-1β was also significantly correlated with HBV DNA (p < 0.05) (Supplementary Table 3).

### Dynamic profile of cytokines/chemokines during antiviral treatment

**Figure 1:**
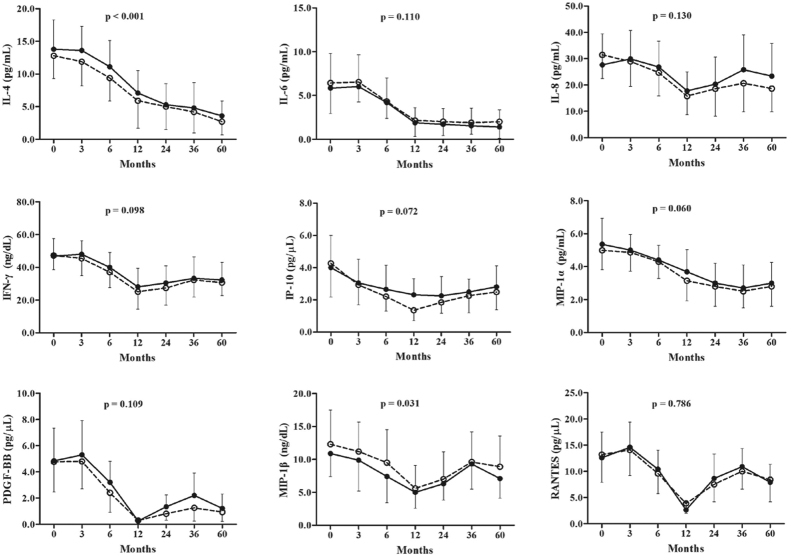
Dynamic change of cytokines/chemokines in patients with CHB during antiviral treatment. In patients with or without HBeAg seroconversion, the levels of cytokines and chemokines declined significantly after 1 year of antiviral treatment. Serum levels of cytokines and chemokines showed opposite trends compared with the first year, except that the levels of IL-4, IL-6, and MIP-1α continued to decrease in both groups.

Longitudinal analysis of each cytokine/chemokine level was carried out at 3, 6, 12, 24, 36 and 60 months after the initiation of ETV therapy (Fig. 1). A significant difference in dynamic changes for each cytokine/chemokine was observed at year 5 of ETV therapy in each group (p < 0.001). Furthermore, the changes over time in IL-4 and MIP-1β levels significantly differed between patients with and without HBeAg seroconversion (both p < 0.05). However, no significant interaction over time for any cytokine/chemokine examined was found. During the first 3 months of antiviral treatment, an increasing trend in levels of IL-6, PDGF-BB and RANTES was observed in both patient groups, while a trend toward increasing levels of IL-8 and IFN-γ was observed exclusively in patients with HBeAg seroconversion. During month 3 to year 1 of treatment, there was an overall trend toward decreasing levels of all the cytokines/chemokines tested in both groups. However, from year 1 to year 5 of treatment, serum levels of cytokines/chemokines showed opposite trends compared with the first year, except for levels of IL-4, IL-6, and MIP-1α, all of which continued to decrease.

Changes in levels of serum cytokines/chemokines in patients with or without HBeAg seroconversion at year 1 and year 5 post-treatment are shown in Table 2. Significant decreases in levels of ALT, AST, HBsAg, HBeAg as well as the nine cytokines/chemokines were found at year 1 post-treatment in patients both with and without HBeAg seroconversion. Similarly, significant decreases in levels of ALT, AST, HBsAg, HBeAg, IL-4, IL-6, IFN-γ, MIP-1α, PDGF-BB, MIP-1β, and RANTES were found after 5 years of treatment in both patient groups, whereas levels of IL-8 and IP-10 at year 5 post-treatment were significantly lower than those at baseline in patients without HBeAg seroconversion, exclusively.

### IP-10 declining associated with year 5 post-treatment in HBeAg seroconversion patients

**Figure 2:**
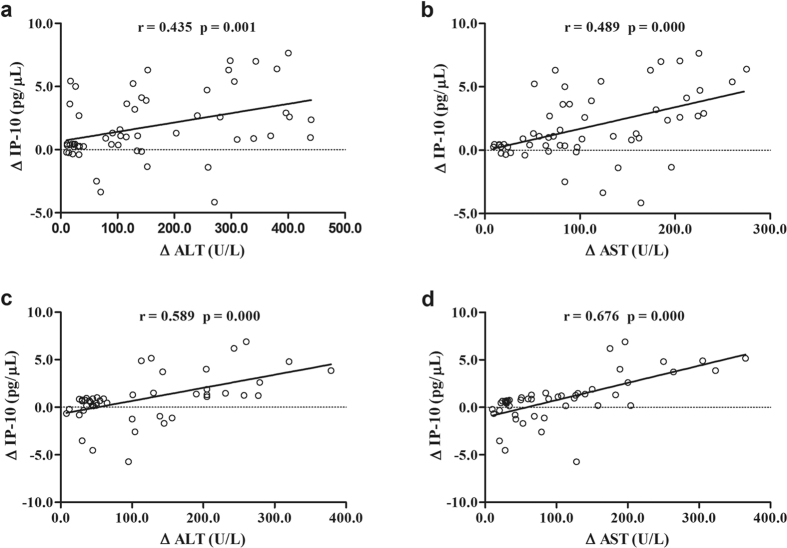
Correlation between the changes of IP-10 (ΔIP-10), ALT (ΔALT) and AST (ΔAST) in CHB patients with ETV treatment. Relationships among the ΔIP-10 with ΔALT and ΔAST during 5 years post-ETV treatment are shown as scatter plots and regression lines. The coefficient r is taken from Spearman’s correlation test. (**a**) and (**b**) Non-HBeAg seroconversion; (**c**) and (**d**) HBeAg seroconversion.

In patients with HBeAg seroconversion, serum IP-10 levels dropped from 4.0 pg/μL at baseline to 2.3 pg/μL at year 1, and remained static at 2.2 pg/μL at year 2, after which the concentration slightly increased to 2.8 pg/μL at year 5. In patients without HBeAg seroconversion, however, serum IP-10 levels dropped from 4.3 pg/μL at baseline to 2.5 pg/μL at month 3, and continued to decrease to 1.3 pg/μL at year 1, after which levels increased to 1.8 pg/μL at year 2 and reached their highest levels of 2.5 pg/μL at year 5 (Fig. 1). The decrease rate of IP-10 levels after 1 year of ETV treatment [(baseline-year1)/baseline] (59.1% vs. 37.1%, p = 0.005) in patients without HBeAg seroconversion was significantly higher than that in patients with HBeAg seroconversion (Table 2). The decrease in IP-10 levels (ΔIP-10) was significantly correlated with a decrease in ALT (ΔALT) and AST (ΔAST) during 5 years of ETV treatment in both groups, respectively (Fig. 2). However, no correlations were observed between ΔIP-10 with decrease in TB, HBsAg, HBeAg, and HBV DNA in either group.

**Figure 3:**
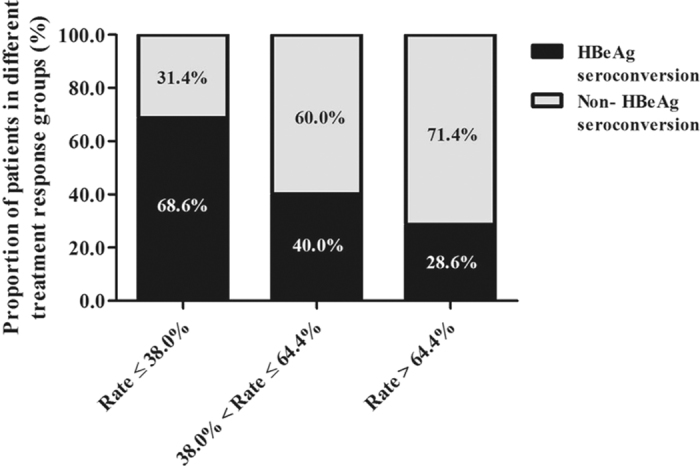
Distribution of chronic hepatitis B patients according to the decrease rate of IP-10 levels after 1 year of ETV treatment. Subjects were sorted into three groups according to tertiles of decrease rate of IP-10 levels.

The decrease rate of IP-10 levels after 1 year of treatment was further arranged in ascending order, and subsequently, subjects were sorted into three groups according to tertiles of the values. Figure 3 shows the treatment response of all 105 patients according to the decrease rate of IP-10 levels after 1 year of ETV treatment. 24 (68.6%), 14 (40.0%) and 10 (28.6%) patients achieved a year 5 HBeAg seroconversion in the low tertile group, middle tertile group and high tertile group, respectively. The proportion of patients who developed HBeAg seroconversion after 5 years of therapy in low tertile group was much higher than those in the other two groups (p < 0.05). Using multivariable Cox logistic regression the low decrease rate of IP-10 levels after 1 year of ETV treatment was an independent predictor of HBeAg seroconversion at year 5 (Hazard ratio [HR] = 0.972, 95% confidence interval [CI] = 0.948–0.996, p = 0.012, Table 3).

**Figure 4:**
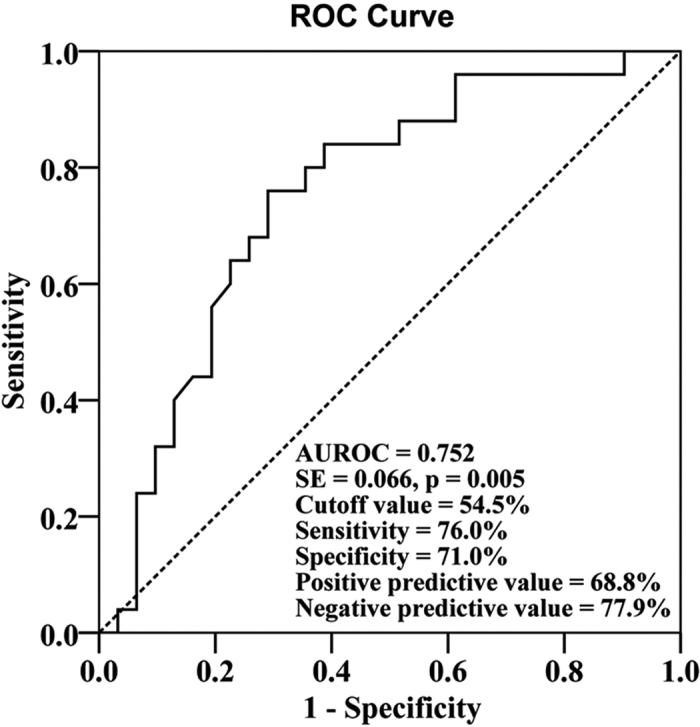
Receiver operator characteristic (ROC) curves. The ROC curves of the decrease rate for IP-10 from initiation of ETV therapy to 1 year post-ETV treatment for differentiating a year 5 HBeAg seroconversion in HBeAg-positive patients.

**Figure 5:**
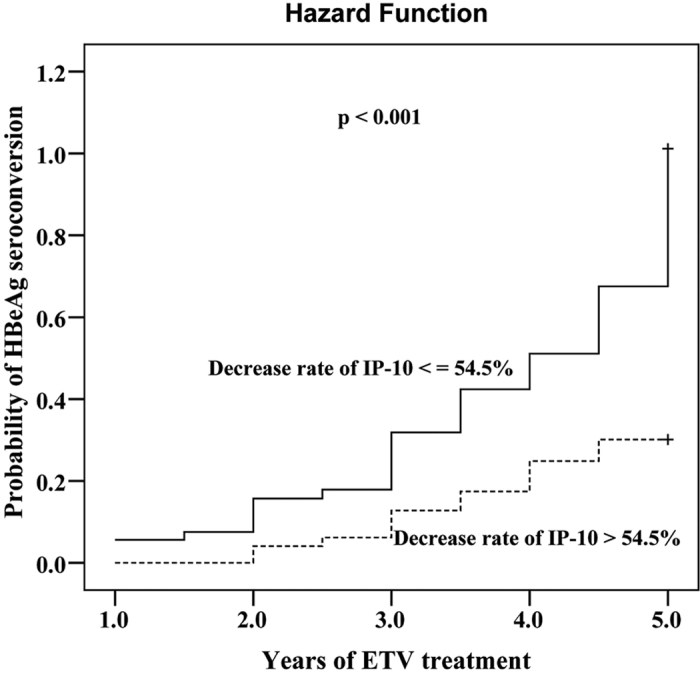
Probability of HBeAg seroconversion in 105 ETV-treated HBeAg-positive CHB patients in relation to decrease rate of IP-10 levels after 1 year of treatment (p < 0.001 by log-rank test).

ROC curves were plotted to define the optimal cutoff values of decrease rate of IP-10 levels after 1 year of treatment for discriminating a year 5 HBeAg seroconversion in HBeAg-positive patients. The area under ROC curve was 0.752 (p = 0.005) with a sensitivity of 76.0% and a specificity of 71.0% under the cutoff value of 54.5% (Fig. 4). When the cutoff value of 54.5% was applied to assess the cumulative rates of HBeAg seroconversion during ETV treatment, patients with decrease rate of IP-10 ≤ 54.5% were found to achieve significantly higher probability of HBeAg seroconversion (HR: 2.977, 95% CI: 1.574–5.631, p = 0.001, Fig. 5).

## Discussion

ETV has significant efficiency and good tolerability in inhibiting HBV and successfully been used to treat various HBV patients^6^. In the present study, after 1 year of ETV antiviral treatment, AST and TB levels in all patients were considered to be normal. ALT levels in all patients in the HBeAg seroconversion group decreased to normal values and HBV DNA in 89.6% (43/48) was also below the detection limit; in the patient cohort without HBeAg seroconversion, there were ten patients (17.5%) whose ALT levels were abnormal, and the percentage of patients whose HBV DNA levels were below the detection limit was 56.1% (32/57), however, the HBV DNA levels in all other patients without HBeAg seroconversion were below 10^5^ IU/mL. After year 5 of ETV antiviral treatment, ALT, AST, and TB, as well as HBV DNA of all patients recovered to normal levels. These results suggest that treatment of ETV can effectively improve the biochemical and virological features of HBV patients and is consistent with the results of previous studies^16^,^17^. Moreover, our results demonstrate that IP-10 has a potential to be an independent prognostic factor for HBeAg seroconversion of the patients with 5 years ETV treatment.

After the initial non-specific immunity response to the HBV virus, specific immunity plays a critical role in fighting against the surviving virus^18^. T lymphocytes are important effector cells in specific immune response and following stimulation of HBV antigens, T cells are classified as killer T cells [mainly CD8+ cytotoxic T lymphocytes (CTLs)] and helper T lymphocytes (Th). CTLs clean the virus both by killing infected cells directly and more importantly, through non-cytolytic effects mediated by cytokines such as IFN-γ and tumour necrosis factor (TNF)-α. Th1 cells mediate cellular immunity through secretion of TNF, IFN-γ, IL-1, IL-12 and other cytokines. While Th1-type immunity dominates, cellular immunity is promoted and the activity of CD8+ T cells is strengthened, thereby facilitating the clearance of viruses. Th2 cells secrete cytokines including IL-4, IL-10, IL-5, IL-6 and IL-13, all of which inhibit Th1 cytokines and mediate humoral immunity^19^,^20^. The Th2 immunity response is associated with the chronicity of HBV infection and it is well known that the imbalance of the Th1/Th2 immune response is reflected by an imbalance of cytokines and is one of the important factors affecting the clearance of viruses which ultimately lead to the chronicity of the infection^21^,^22^. Our study showed that concentrations of cytokines in patients with CHB were significantly higher than normal control subjects, which implies that after the infection of HBV, the immune system is activated and the Th1/Th2 balance is re-established through high cytokine levels^20^,^23^.

Chemokines interact with their various receptors help to regulate the migration of immune cells and play a very important role in the body’s immune response. Excessive or abnormal expression of chemokines can lead to excessive inflammation and studies have shown inflammatory infiltration of liver tissue is accompanied by an increased expression of chemokines^24^,^25^. Chemokines mediate the recognition, adhesion, and infiltration of Th1 lymphocytes. A Previous study suggested that MIP-1α and MIP-1β could promote liver fibrosis and mediate the infiltration of T cells with high expression of CCR5 in the portal area, leading to an imbalance in the immune response and the persistent infection of HBV^26^. Our study indicated that serum levels of MIP-1β were positively correlated with the replicative activity of HBV which further supports the theory that MIP-1β is associated with persistent HBV infection and may be of therapeutic potential target in anti-HBV treatment. The role of MIP-1β in chronic HBV infection can be elucidated by investigating the correlation of MIP-1β and the stratification of the HBV DNA load. IP-10 selectively induces the chemotaxis of CXC3 receptor expressing Th1 cells which enhances the Th1 response and increases the secretion of IFN-γ, all of which ultimately induces and maintains the chronic inflammatory response^27^. Correlation analysis of the levels of cytokines and chemokines with clinical parameters at baseline in our study indicated that the levels of these factors were positively correlated with the levels of ALT, AST and TB. Therefore, to a certain extent, the serum levels of these cytokines and chemokines reflect the inflammation activity of chronic HBV infection and can serve as indicators measuring the severity of liver damage.

Our study found that relative to the baseline level, the levels of cytokines and chemokines changed significantly after 5 years post-ETV treatment in both groups studied. Interestingly, levels of the various cytokines and chemokines tested were significantly reduced after 1 year of antiviral treatment. This suggests that after HBV replication was effectively inhibited by ETV, the immune response of the patients also underwent significant changes^28^. Results suggest that the inflammatory response decreased, damage of liver was reduced and the ability of the body to the clear the virus gradually recovered. However, the detailed mechanisms associated with the reduction in inflammation after 1 year post-ETV antiviral treatment remains unclear. We propose that the reduction of the inflammatory response may be associated with the decrease of the virus load after treatment and mitigation of antigen stimulation^9^. Moreover, immunological tolerance may also play a role. Since the elimination of HBV needs HBV-specific T lymphocytes, if the ability of HBV antigen-specific CD8+ T cells could not effectively clear HBV, immune tolerance to HBV may occur. This may also explain why the seroconversion rate is usually unsatisfactory following nucleotide analog treatment, as the immune response has yet to be fully restored^29^. Furthermore, during last 4 years of the 5 year post-ETV treatment, only IL-4, IL-6 and MIP-1α continued to decrease, while other factors increased to varying degrees. This implies the host immune response varies with the stages of chronic HBV infection and exhibits diverse processes during the antiviral treatment^30^.

IP-10 is a member of the non-ELR (glutamic-leucine-arginine) motif CXC chemokine family. A previous study showed that expression of IP-10 is upregulated in chronic HBV infection. IP-10 mediates the inflammation response and is associated with the chronicity of hepatitis B^27^. Our results showed that decreases in serum IP-10 levels after 5 years post-ETV treatment was significantly positively correlated with a decrease in ALT and AST, suggesting that the levels of IP-10 dynamically reflected the severity of the damage of hepatocytes caused by active inflammation of HBV and IP-10 may be a valuable marker for evaluating the process of chronic HBV infection. Our study also showed the levels of IP-10 were independent of the replicative activity of HBV which was consistent with the opinion that the replicative level of HBV is not always positively correlated with the severity of liver inflammation^31^.

Wong *et al.* showed that pre-treatment levels of IP-10, as well as the change of IP-10 during the treatment, was irrelevant to the HBsAg seroconversion and that only post-treatment levels of IP-10 were associated with the HBsAg seroconversion^32^. Another study suggested that a high level of IP-10 before the treatment of interferon facilitated the negative conversion of serum HBeAg^33^. In the current study, we did not find any association between baseline levels of IP-10 and HBeAg seroconversion with 5 year antiviral treatment, however, we did find that the degree of IP-10 decrease was closely correlated with HBeAg seroconversion. Specifically, after 1 year of treatment, the degree of IP-10 decrease was significantly lower in the HBeAg seroconversion group than in the HBeAg non-seroconversion group. Our study suggests that during ETV treatment, maintaining a high level of serum IP-10 is paramount for long-term HBeAg seroconversion. The degree of IP-10 decrease after 1 year post-ETV treatment is probably an independent predictive factor for HBeAg seroconversion after 5 years post-treatment. A previous study indicated that IP-10 in HBV-infected patients was predominantly produced by hepatocytes, and was seldom produced in any other organ analyzed^34^. Therefore, compared to other cytokines which can be produced by multiple organs, such as TNF-α, IP-10 is more likely to be a highly-specific and sensitive indicator to predict inflammatory injury of liver. Furthermore, dynamically monitoring the serum levels of IP-10 in HBV-infected patients during antiviral treatment is helpful for predicting the prognostic prediction of HBV patients and long-term efficacy of this treatment.

In conclusion, our study indicates that ETV antiviral treatment can effectively control HBV replication and improve liver function. Additionally, IL-4 and MIP-1β may play an important role in HBeAg seroconversion, although further studies are needed to determine the role of these cytokines in this process. Finally, the change in IP-10 levels during ETV treatment is closely associated with long-term HBeAg seroconversion and the decrease in IP-10 maybe an independent predictive factor for HBeAg seroconversion after 5 years post-ETV treatment.

Although patients with and without HBeAg seroconversion had been matched in several important clinical parameters (sex, age, HBV genotype and cirrhosis status), there might still be unmeasured confounders, which is one of the study’s limitations. We only found a significant association between PDGF-BB level and cirrhosis before treatment, which may be related with exclusion of patients with decompensated liver cirrhosis in our study, but this finding should be interpreted with caution due to the relatively small number of patients who had both cytokines/chemokines measurements and liver biopsies. Furthermore, a study should be carried out to evaluate the histological findings and the relationship between cytokines/chemokines and HBV-induced cirrhosis in a large cohort of CHB patients.

## Methods

### Subject enrollment

One hundred and nineteen consecutive treatment- naïve CHB patients who were treated orally with 0.5 mg of ETV daily at the Department of Infectious Diseases of the First affiliated Hospital of Zhejiang University, Hangzhou, China from April 2007 to January 2015 were recruited for the study. The definition of CHB was determined according to the diagnostic standard of the National Program for Prevention and Treatment of Viral Hepatitis^35^,^36^. Patients were administered ETV based on the following criteria: (1) all patients were hepatitis B surface antigen (HBsAg) positive for at least 6 months before treatment; (2) patient HBV DNA levels were >20,000 IU/mL; (3) patients had increased ALT levels, but these levels were ≤10 × upper limit of normal [ULN] or histological levels found in chronic active hepatitis; (4) the ETV treatment was naïve; (5) patients were aged between 18 and 65 years in the enrolling year. Exclusion criteria for patients with CHB were (1) co-infection with either hepatitis A, hepatitis C, or hepatitis D viruses or human immunodeficiency virus; (2) alcohol- or drug-induced autoimmune liver diseases, other acquired or inherited causes of liver disease, decompensated liver cirrhosis or (3) other severe diseases. A detailed description is shown in our previous report^37^.

The diagnosis of liver fibrosis or cirrhosis before antiviral treatment was based on liver biopsies. All histological specimens from the eligible patients were evaluated by an experienced pathologist. Histological changes were evaluated according to the Metavir scoring scheme^38^. Healthy controls (n = 68) with normal serum ALT levels and undetectable HBsAg were enrolled from the Health Examination Center of the First affiliated Hospital of Zhejiang University and were matched for age and gender with CHB patients.

Patients were followed-up and blood samples were collected every 3 months during the first year and every 6 months thereafter for clinical assessments including liver biochemical tests, hematologic examinations, serological hepatitis B markers and serial HBV DNA levels. HBeAg seroconversion was defined as HBeAg loss with formation of anti-HBe antibody (HBeAb). This study was conducted in agreement with the ethical principles of the Declaration of Helsinki, and the study protocol was approved by the Ethics Committee of the First Affiliated Hospital of Zhejiang University. Written informed consent was obtained from each patient before the initiation of the study.

### Laboratory assays

Blood samples of patients who had fasted were obtained from an antecubital vein and used for subsequent analyses. Serum was isolated from the peripheral blood and stored at −80 °C until the concentrations of cytokines were measured. Levels of serum albumin (ALB), ALT, aspartate aminotransferase (AST), and total bilirubin (TB) were determined using a Hitachi 7600 analyzer (Hitachi Ltd., Tokyo, Japan) with dedicated reagents (Roche Diagnostics, Mannheim, Germany). HBV genotype was determined by nested PCR using type-specific primers^39^. Serum HBV DNA levels were assayed using COBAS Amplicor/COBAS TaqMan HBV test (Roche Diagnostics, Indianapolis, IN, USA) with a detection limit of 20 IU/mL according to the manufacturer’s instructions. HBV serologic tests for HBsAg, HBeAg, anti-HBs antibody, and HBeAb were conducted by enzyme immunoassay (AxSYM; Abbott Laboratories, Abbott Park, IL, USA). Four cytokines (IL-4, IL-6, IL-8, and interferon [IFN]-γ) and five chemokines (macro-phage inflammatory protein [MIP]-1α, MIP-1β, regulated on activation normal T cell expressed and secreted [RANTES], platelet derived growth factor-BB [PDGF-BB], and interferon-inducible protein 10 [IP-10]) were determined retrospectively in stored serum samples, and measured using a Luminex 200 multiplexing instrument (EMD Millipore, Billerica, MA, USA) with a Bio-Plex Reagent Kit, Diluent Kit, and human Grp I Cytokine 27-Plex Panelv (Bio-Rad, Hercules, CA, USA) according to the manufacturer’s instructions.

### Statistical analysis

The data were expressed as the mean and standard deviation for continuous variables, and as absolute and relative frequencies for categorical variables. Continuous variables were compared using Student’s t test or Mann–Whitney U test for two independent groups and a paired t test for two related groups. The χ^2^ test or Fisher exact test was used to compare the difference in proportions between the treatment groups. Spearman rank correlation was used to evaluate the relationship between parameters. To determine independent predictive factors for HBeAg seroconversion, variables with a probability (p) value < 0.1 in univariate analysis were included in a multivariate Cox proportional hazard regression model. The accuracy of decreases in serum cytokines to predict HBeAg seroconversion was assessed using the receiver operating characteristic curve (ROC). Kaplan-Meier curves were used for the estimation of outcome rates over time. All statistical tests were performed using SPSS, version 16.0 (SPSS Inc., Chicago, IL, USA) and p < 0.05 was considered statistically significant.

## Additional Information

**How to cite this article**: Guo, R. *et al.* Slow reduction of IP-10 Levels predicts HBeAg seroconversion in chronic hepatitis B patients with 5 years of entecavir treatment. *Sci. Rep.* **6**, 37015; doi: 10.1038/srep37015 (2016).

**Publisher’s note**: Springer Nature remains neutral with regard to jurisdictional claims in published maps and institutional affiliations.

## Supplementary Material

Supplementary Information

## Figures and Tables

**Table 1:** Baseline demographic and clinical characteristics of the enrolled participants.

Variable	Chronic hepatitis B					
	Non- HBeAg seroconversion (n = 57)	HBeAg seroconversion (n = 48)	Overall (n = 105)	Healthy control (n = 68)	p^a^	p^b^
Sex (male/female)	45/12	35/13	80/25	46/22	0.470	0.217
Age (years)	36.1 (10.0)	34.5 (10.3)	36.5 (10.1)	35.6 (11.3)	0.565	0.835
HBV genotype (B/C/D)	28/22/7	21/18/9	49/40/16	—	0.642	—
Liver biopsy [N (%)]	37 (64.9)	34 (70.8)	71 (67.6)	—	0.518	—
Metavir score	2.2 (1.1)	2.1 (1.2)	2.2 (1.1)	—	0.479	—
Cirrhosis [N (%)]	6 (16.2)	7 (20.6)	13 (18.3)	—	0.634	—
TB (μmol/L)	15.2 (9.3)	14.7 (4.7)	14.9 (7.1)	9.4 (2.5)	0.460	0.003
ALB (g/L)	45.8 (3.2)	46.8 (3.1)	46.1 (3.4)	48.5 (2.3)	0.270	0.034
ALT (U/L)	240.5 (152.9)	161.2 (109.6)	204.1 (133.6)	15.6 (9.4)	0.164	0.000
AST (U/L)	136.5 (75.7)	131.5 (110.7)	137.2 (94.9)	17.2 (3.4)	0.562	0.000
HBsAg (× 10^3^ IU/mL)	44.4 (39.7)	7.0 (9.5)	23.2 (31.6)	NA	0.001	NA
HBeAg (PEIU/mL)	187.1 (82.8)	77.9 (105.8)	140.1 (109.0)	NA	0.012	NA
HBV DNA (log_10_ IU/mL)	8.1 (1.4)	6.8 (1.0)	7.3 (1.4)	NA	0.002	NA

All data are mean (standard deviation) exception indication; NA, not available.

^a^Non-HBeAg seroconversion vs. HBeAg seroconversion.

^b^Overall vs. Healthy control.

**Table 2:** Comparisons of changes in serum cytokines/chemokines in patients with or without HBeAg seroconversion at year 1 and year 5 post-ETV treatment.

Variable	Non- HBeAg seroconversion (n = 57)							HBeAg seroconversion (n = 48)							p^c^	p^d^
	Baseline	Year 1	Year 5	p^a^	p^b^	Rate 1 (%)	Rate 2 (%)	Baseline	Year 1	Year 5	p^a^	p^b^	Rate 1 (%)	Rate 2 (%)		
Clinical data																
TB (mg/dL)	15.2 (9.3)	12.3 (3.9)	9.4 (3.9)	0.286	0.070	1.5 (39.9)	21.8 (45.2)	14.7 (4.7)	13.5 (4.3)	9.9 (3.4)	0.607	0.062	0.2 (27.6)	23.7 (42.3)	0.902	1.000
ALT (U/L)	240.5 (152.9)	22.5 (11.0)	20.8 (6.8)	0.001	0.001	80.6 (24.4)	81.5 (19.2)	161.2 (109.6)	19.9 (4.6)	18.9 (6.7)	0.002	0.001	74.3 (28.1)	81.7 (12.2)	0.156	0.505
AST (U/L)	136.5 (75.7)	21.1 (3.6)	19.5 (3.3)	0.001	0.000	75.6 (22.1)	77.4 (21.0)	131.5 (110.7)	22.8(4.9)	19.4 (5.8)	0.001	0.003	68.1 (17.6)	76.1 (14.7)	0.140	0.469
HBsAg (×10^3^ IU/mL)	44.4 (39.7)	4.1 (5.4)	7.6 (12.5)	0.010	0.043	86.9 (14.4)	70.0 (39.5)	7.0 (9.5)	1.7 (1.7)	2.9 (2.2)	0.019	0.021	13.3 (94.6)	15.3 (72.3)	0.074	0.099
HBeAg (PEIU/mL)	187.1 (82.8)	29.5 (58.7)	0.6 (0.5)	0.004	0.004	83.2 (28.3)	99.6 (0.4)	77.9 (105.8)	7.1 (23.4)	0.0 (0.0)	0.020	0.032	88.6 (12.8)	97.7 (5.6)	0.537	0.317
HBV DNA (P/N, n)	57/0	25/32	0/57	0.000	0.000	—	—	48/0	5/43	0/48	0.000	0.000	—	—	—	—
Cytokines/chemokines																
IL-4(pg/mL)	12.8 (3.5)	5.9 (4.2)	2.7 (2.0)	0.000	0.000	51.7 (35.2)	77.2 (18.5)	13.8 (4.5)	7.1 (3.4)	3.6 (2.3)	0.000	0.000	45.9 (30.8)	72.0 (19.3)	0.550	0.291
IL-6(pg/mL)	6.4 (3.4)	2.1 (1.5)	2.0 (1.3)	0.000	0.000	65.0 (22.1)	62.3 (30.6)	5.8 (2.9)	1.9 (1.6)	1.4 (1.3)	0.011	0.006	55.1 (38.7)	65.5 (33.0)	0.854	0.818
IL-8(pg/mL)	31.4 (9.0)	15.7 (7.0)	18.7 (8.9)	0.000	0.006	46.5 (26.2)	33.1 (41.2)	27.7 (11.8)	17.7 (7.2)	23.3 (12.5)	0.005	0.411	29.2 (31.6)	10.7 (63.1)	0.066	0.198
IFN-γ (ng/dL)	47.3 (8.7)	25.1 (10.8)	30.6 (7.8)	0.000	0.000	46.0 (31.1)	33.6 (16.8)	46.9 (10.7)	28.1 (11.3)	32.3 (10.9)	0.002	0.021	36.8 (28.1)	26.5 (37.8)	0.421	0.679
IP-10 (pg/μL)	4.3 (2.1)	1.3 (0.6)	2.5 (1.1)	0.001	0.028	**59.1 (20.9)**	28.4 (44.6)	4.0 (2.0)	2.3 (1.0)	2.8 (1.3)	0.003	0.162	**37.1 (25.0)**	8.7 (75.9)	**0.005**	0.431
MIP-1α (pg/mL)	5.0 (1.2)	3.1 (1.2)	2.8 (1.2)	0.000	0.007	36.7 (30.0)	43.7 (25.2)	5.4 (1.6)	3.7 (1.3)	3.0 (1.3)	0.002	0.000	29.3 (29.5)	42.5 (23.2)	0.613	0.946
PDGF-BB (pg/μL)	4.8 (2.3)	0.3 (0.2)	0.9 (0.7)	0.001	0.004	83.8 (24.7)	20.8 (144.6)	4.8 (2.5)	0.3 (0.1)	1.2 (1.1)	0.004	0.013	78.3 (29.1)	15.2 (133.4)	0.963	0.435
MIP-1β (ng/dL)	12.3 (5.2)	5.6 (3.5)	8.9 (4.7)	0.000	0.018	51.8 (30.7)	17.2 (60.5)	10.9 (3.5)	5.0 (2.4)	7.1 (3.0)	0.000	0.019	45.3 (26.7)	19.2 (45.9)	0.251	0.713
RANTES (pg/μL)	13.2 (5.3)	3.9 (1.9)	7.6 (3.4)	0.000	0.001	71.3 (9.2)	36.0 (34.7)	12.6 (4.9)	2.6 (1.5)	6.4 (3.7)	0.000	0.002	75.1 (16.6)	41.5 (34.0)	0.210	0.734

All data are mean (standard deviation) exception indication; Rate 1 = (Baseline –Year 1)/Baseline; Rate 2 = (Baseline –Year 5)/Baseline.

^a^Baseline vs. year 1.

^b^Baseline vs. year 5.

^c^Rate 1 (non-HBeAg seroconversion) vs. Rate 1 (HBeAg seroconversion).

^d^Rate 2 (non-HBeAg seroconversion) vs. Rate 2 (HBeAg seroconversion); P: positive; N: negative.

**Table 3:** Cox regression analyses of decrease rate of clinical parameters after 1 year of treatment for prediction of HBeAg seroconversion at year 5.

Variables	HR (95% CI)	p value
Univariate analysis		
Sex (Femal vs. Male)	2.030 (0.679–6.068)	0.205
Age	0.991 (0.941–1.044)	0.730
HBV genotype (Non-C vs. C)	0.531 (0.305–1.146)	0.240
Cirrhosis (Yes vs. No)^a^	1.353 (0.537–3.410)	0.522
Metavir score^a^	1.003 (0.702–1.431)	0.980
TB	0.999 (0.983–1.015)	0.889
ALT	0.998 (0.980–1.016)	0.811
AST	0.992 (0.968–1.018)	0.548
HBsAg	0.472 (0.219–1.018)	0.056
HBeAg	1.014 (0.979–1.051)	0.439
HBV DNA^a^	0.688 (0.552–0.859)	0.004
IL-4	0.997 (0.981–1.012)	0.666
IL-6	0.987 (0.970–1.004)	0.140
IL-8	0.986 (0.970–1.002)	0.078
IFN-γ	0.992 (0.974–1.010)	0.385
IP-10	0.976 (0.959–0.994)	0.008
MIP-1α	0.996 (0.979–1.013)	0.654
PDGF-BB	0.997 (0.980–1.015)	0.781
MIP-1β	0.997 (0.980–1.014)	0.753
RANTES	1.016 (0.973–1.060)	0.482
Multivariate analysis		
HBsAg	0.481 (0.200–1.105)	0.084
HBV DNA^a^	0.686 (0.430–1.097)	0.116
IL-8	0.993 (0.981–1.006)	0.314
IP-10	0.972 (0.948–0.996)	0.012

^a^Liver biopsy or HBV DNA levels before ETV therapy; HR: hazard ratio; CI: confidence interval.
