# Supplementary material for: Slow reduction of IP-10 Levels predicts HBeAg seroconversion in chronic hepatitis B patients with 5 years of entecavir treatment
Source: Sci Rep. 2016 Nov 15;6:37015. doi: 10.1038/srep37015 (PMC5109480; doi:10.1038/srep37015)
Supplement: Supplementary Information [file srep37015-s1.pdf]

Slow reduction of IP-10 levels predicts HBeAg seroconversion in chronic hepatitis B patients with 5 years of entecavir treatment

Renyong Guo[1, †], Hejun Mao[2, †], Xiao Hu[1], Nengneng Zheng[3], Dong Yan[4], Jianqin He[4] and Jiezuan Yang[4,*]

[1]Department of Laboratory Medicine, First Affiliated Hospital, College of Medicine, Zhejiang University; Key Laboratory of Clinical In Vitro Diagnostic Techniques of Zhejiang Province, Hangzhou, China

[2] Maternity and Child Health Care Hospital of Xiacheng District, Hangzhou, China

[3]Department of Gynecology and Obstetrics, Tongde Hospital of Zhejiang Province, Hangzhou, China

[4]State Key Laboratory for Diagnosis and Treatment of Infectious Diseases; Collaborative Innovation Center for Diagnosis and Treatment of Infectious Diseases; the First Affiliated Hospital, College of Medicine, Zhejiang University, Hangzhou, China

[†]Renyong Guo and Hejun Mao contributed equally to this work

*Corresponding Author: Jiezuan Yang, PhD

State Key Laboratory for Diagnosis and Treatment of Infectious Diseases,

No. 79 Qingchun Road, Hangzhou, Zhejiang, 310003, China.

Supplementary Table 1. Baseline serum cytokines/chemokines in chronic hepatitis B patients and healthy subjects.

Variable	Chronic hepatitis B			Healthy control (n = 68)	p [a]	p [b]
	Non-HBeAg seroconversion (n = 57)	HBeAg seroconversion (n = 48)	Overall (n = 105)			
IL-4 (pg/mL)	12.8 (3.5)	13.8 (4.5)	13.3 (4.0)	6.3 (4.7)	0.713	0.000
IL-6 (pg/mL)	6.4 (3.4)	5.8 (2.9)	6.1 (3.1)	2.5 (2.6)	0.382	0.001
IL-8 (pg/mL)	31.4 (9.0)	27.7 (11.8)	29.5 (10.5)	17.4 (8.9)	0.232	0.001
IFN-γ (ng/dL)	47.3 (8.7)	46.9 (10.7)	47.1 (10.1)	35.4 (13.9)	0.730	0.009
IP-10 (pg/ μL)	4.3 (2.1)	4.0 (2.0)	4.1 (2.2)	2.8 (2.0)	0.600	0.003
MIP-1α (pg/mL)	5.0 (1.2)	5.4 (1.6)	5.2 (1.4)	3.5 (2.0)	0.565	0.002
PDGF-BB (pg/ μL)	4.8 (2.3)	4.8 (2.5)	4.8 (2.5)	0.5 (0.3)	0.679	0.000
MIP-1β (ng/dL)	12.3 (5.2)	10.9 (3.5)	11.6 (4.3)	8.3 (4.0)	0.129	0.020
RANTES (pg/ μL)	13.2 (5.3)	12.6 (4.9)	12.9 (5.1)	4.3 (1.9)	0.635	0.001

All data expressed as mean (standard deviation). [a], No HBeAg seroconversion vs. HBeAg seroconversion; [b], Overall vs. Healthy control.

Supplementary Table 2. Baseline demographic characteristics of the enrolled participants according to fibrosis status

Variable	Non- cirrhosis (Metavir score ≤ 3, n = 58)	Cirrhosis (Metavir score = 4, n = 13)	p
Sex (male/female)	44/14	11/2	0.495
Age (years)	35.5 (9.4)	38.7 (10.3)	0.089
HBV genotype (B/C/D)	31/19/8	4/6/3	0.326
TB (μmol/L)	14.2 (6.5)	16.9 (8.8)	0.163
ALB (g/L)	46.8 (3.1)	45.1(4.4)	0.212
ALT (U/L)	196.5 (123.4)	218.9 (95.0)	0.188
AST (U/L)	123.8 (97.5)	165.2 (104.8)	0.045
HBsAg ($\times 10^3$ IU/mL)	27.6 (35.8)	10.8 (7.9)	0.063
HBeAg (PEIU/mL)	165.5 (108.9)	123.6 (92.1)	0.230
HBV DNA ($\log_{10}$ IU/mL)	7.5 (1.5)	7.1 (0.9)	0.152
IL-4 (pg/mL)	13.4 (3.1)	13.2 (5.1)	0.644
IL-6 (pg/mL)	5.6 (3.9)	6.9 (4.7)	0.051
IL-8 (pg/mL)	29.7 (9.6)	30.4 (12.3)	0.769
IFN-γ (ng/dL)	45.3 (11.5)	49.4 (18.3)	0.113
IP-10 (pg/ μL)	3.9 (2.5)	4.4 (3.1)	0.226
MIP-1α (pg/mL)	5.1 (1.0)	5.3 (1.9)	0.370
PDGF-BB (pg/ μL)	3.6 (3.5)	5.2 (4.4)	0.048
MIP-1β (ng/dL)	11.9 (4.3)	10.8 (6.7)	0.201
RANTES (pg/ μL)	14.3 (5.0)	12.5 (5.8)	0.138

All data expressed as mean (standard deviation).

Supplementary Table 3. Correlation between cytokines/chemokines and clinical parameters at baseline.

	IL-4	IL-6	IL-8	IFN-γ	IP-10	MIP-1α	PDGF-BB	MIP-1β	RANTES
TB	0.231	0.281	0.255	0.197	0.262	0.225	0.205	0.172	0.261
ALB	-0.002	-0.067	-0.011	0.020	0.057	0.028	-0.025	0.126	0.063
ALT	0.486**	0.438**	0.434**	0.365*	0.444**	0.408**	0.465**	0.303*	0.473**
AST	0.548**	0.465**	0.500**	0.395**	0.557**	0.464**	0.517**	0.321*	0.531**
HBsAg	-0.129	0.062	0.131	-0.088	0.067	-0.199	0.067	-0.006	0.073
HBeAg	-0.307	-0.321	-0.151	-0.299	0.130	-0.261	-0.184	0.059	-0.257
HBV DNA	0.114	0.021	0.105	-0.047	0.118	0.067	0.023	0.339*	-0.042

Spearman's rank correlation coefficients between cytokines/chemokines and clinical parameters. *, $p < 0.05$; **, $p < 0.01$.
